# Modeling Alzheimer’s Disease in *Caenorhabditis elegans*

**DOI:** 10.3390/biomedicines10020288

**Published:** 2022-01-26

**Authors:** Javier Alvarez, Pilar Alvarez-Illera, Jaime Santo-Domingo, Rosalba I. Fonteriz, Mayte Montero

**Affiliations:** Departamento de Bioquímica y Biología Molecular y Fisiología, Facultad de Medicina, Unidad de Excelencia Instituto de Biología y Genética Molecular (IBGM), Universidad de Valladolid and CSIC, Ramón y Cajal, 7, E-47005 Valladolid, Spain; pilar_alvill@hotmail.com (P.A.-I.); jaime.santo-domingo@uva.es (J.S.-D.); rfonteri@ibgm.uva.es (R.I.F.); mmontero@ibgm.uva.es (M.M.)

**Keywords:** Alzheimer’s, *C. elegans*, β-amyloid, amyloid precursor protein, tau protein, presenilin, new therapies

## Abstract

Alzheimer’s disease (AD) is the most frequent cause of dementia. After decades of research, we know the importance of the accumulation of protein aggregates such as β-amyloid peptide and phosphorylated tau. We also know that mutations in certain proteins generate early-onset Alzheimer’s disease (EOAD), and many other genes modulate the disease in its sporadic form. However, the precise molecular mechanisms underlying AD pathology are still unclear. Because of ethical limitations, we need to use animal models to investigate these processes. The nematode *Caenorhabditis elegans* has received considerable attention in the last 25 years, since the first AD models overexpressing Aβ peptide were described. We review here the main results obtained using this model to study AD. We include works studying the basic molecular mechanisms of the disease, as well as those searching for new therapeutic targets. Although this model also has important limitations, the ability of this nematode to generate knock-out or overexpression models of any gene, single or combined, and to carry out toxicity, recovery or survival studies in short timeframes with many individuals and at low cost is difficult to overcome. We can predict that its use as a model for various diseases will certainly continue to increase.

## 1. Introduction

Alzheimer’s disease is a chronic and irreversible neurodegenerative disease that constitutes approximately 80% of dementia cases and mainly affects the population over 65 years of age. It is characterized by a progressive loss of cognitive function with severe memory impairment and impaired thinking and social skills, which together make daily living activities considerably more difficult or even impossible and lead to dependence [1,2].

From a neuropathological point of view, Alzheimer’s disease is characterized by the presence of neurofibrillary tangles (NFTs) and extracellular insoluble amyloid plaques, accompanied by neuronal damage and death mainly in the cerebral cortex and hippocampus, brain regions critical for learning and memory. The main constituent of NFTs is a hyperphosphorylated form of tau protein, a phosphoprotein that promotes tubulin assembly on microtubules and helps stabilize their structure. On the other hand, extracellular amyloid plaques are composed mainly of amyloid (Aβ) peptide. Aβ accumulates in extracellular plaques and then there is uptake by endocytosis of these neurotoxic oligomers. This process induces tau phosphorylation and its aggregation into NFTs, as well as other toxicity phenomena, including ER stress, alterations of Ca^2+^ homeostasis, mitochondrial dysfunction, neuroinflammation and neuronal death [1,2].

The accumulation of the Aβ peptide is the result of sequential enzymatic processing of the human amyloid precursor protein (APP) by enzymes called secretases, which are proteases responsible for cleaving this protein. APP is a glycoprotein with a single transmembrane segment that can undergo sequential proteolytic processing through two different pathways, the α- or β-secretase pathways, each with functionally distinct results. Initial cleavage by α- or β-secretase releases an N-terminal soluble extracellular fragment termed sAPPα or sAPPβ, respectively. Subsequent in-membrane cleavage of the C-terminal fragment by the γ-secretase complex releases the APP intracellular domain (AICD). Initial cleavage by α-secretase occurs within the Aβ domain, thus preventing the generation of toxic Aβ peptides after cleavage by γ-secretase. In contrast, when γ-secretase acts on the C-terminal fragment generated by β-secretase, in addition to AICD being generated intracellularly, Aβ peptides are formed, mainly inside endosomes, and they are then released to the extracellular side. This peptide of 37 to 49 amino acids in length has a tendency to aggregate, forming amyloid plaques, is neurotoxic and is believed to be central to the pathogenesis of AD [1,2,3].

Therefore, the pathogenesis of AD often involves increased APP processing by the β-γ-secretase pathway. The catalytic subunit of γ-secretase is constituted by the presenilin protein, and in mammals there are 2 homologous proteins with this function, PSEN1 and PSEN2. Finally, the γ-secretase cleavage site is not always the same. Typically, 90% of the Aβ generated consists of 40 amino acids (Aβ40) and the other 10% consists of 37 to 49 amino acids, with the form of 43 amino acids (Aβ43) being the most abundant. The latter peptides are more easily aggregated and in certain mutations responsible for familial AD there is an increase in the proportion of these fragments, which are assumed to be mainly responsible for the neurotoxic effect [1,2,3].

AD is classified into early-onset (EOAD) and late-onset (LOAD). EOAD usually clinically develops before 60 years, while LOAD or sporadic AD appears after 65 years and is the most frequent, representing more than 95% of all AD cases. EOAD are in some cases due to highly penetrant autosomal dominant mutations in a number of genes, including the amyloid-β-protein precursor (APP), presenilin 1 (*PSEN1*) or presenilin 2 (*PSEN2*) genes. In these cases we speak of familial AD (FAD), which constitutes less than 2% of AD cases [4]. Therefore, FAD is always due to mutations in the Aβ generation process, either in the substrate (*APP*) or in the protease (presenilin). Mutations in these genes also increase the proportion of the Aβ42 form. On the other hand, LOAD cases are associated with less penetrant genetic variants. The main variant is the ε4 allele of the apolipoprotein E (APOE) gene, which can increase the risk of AD by 5- to 20-fold, depending on the number of ε4 alleles present [1,2].

## 2. Models to Study Alzheimer’s Disease

Alzheimer’s disease can be considered a truly human disease because it directly affects the cognitive functions of our species. However, the ethical and operational limitations imposed by research in humans have led to the generation of a whole series of animal models in which to study both the mechanisms of the disease and the effects of different treatments. Given that there are mutations that are associated with the appearance of FAD, models have been generated, for example, using transgenic mice with similar mutations. Other models have been developed with mice treated with pharmacological or chemical agents to induce the appearance of the lesions. In addition, models have been generated in many other mammals, including rats, dogs and rabbits, and in some cases also non-human primates [5,6,7].

These models have made it possible to obtain a great deal of information, but they have important limitations, such as ethical problems, their high cost, a very long study time, as they are animals with a long life span, and the small number of individuals studied, with the consequent statistical difficulty to obtain significant results. For these reasons, smaller model organisms have been developed, with shorter lifespans and that are easier to maintain in large numbers. Although it is more difficult to find a pathological parallel between the symptoms in humans and the effects found in these models, they have been very useful for studying the pathophysiological mechanisms of, for example, the formation of Aβ or tau deposits, and the effects of possible treatments.

Among the small organism models, the most commonly used have been flies (*Drosophila melanogaster*), zebrafish (*Danio rerio*) and worms (*Caenorhabditis elegans*). These models each possess their differential characteristics, advantages and limitations (reviewed in [6,7,8,9,10]). None of them manages to reproduce all the pathophysiological changes that occur in the life of a human being, and neither do the mammalian models, but they still show parallels in various age-related phenomena, such as loss of muscle strength or alterations in locomotion. In this review we will discuss the *C. elegans* model, its characteristics, the Alzheimer’s disease models it has allowed us to generate and the information it has provided, both in terms of disease mechanisms and possible treatments.

## 3. Genes Implicated in Alzheimer’s Disease: Homologs in *C. elegans*

Many human genes possess orthologues in *C. elegans*. Despite the distance on the evolutionary scale, comparative proteomics data indicate that 83% of the *C. elegans* proteome has human homologous genes [11]. A recent study has also shown that 53% of the human coding genome has recognizable orthologues in the worm [12]. In the specific case of the genes involved in Alzheimer’s disease, most of them also possess orthologues in *C. elegans*. As for genes in which mutations are capable of producing EOAD, both APP and presenilins possess orthologues in *C. elegans* (Table 1). On the other hand, genome-wide association studies (GWAS) have identified around 50 genes that have alleles associated with an increased tendency to LOAD [13], and most of these genes also have orthologues in *C. elegans*. Table 1 shows the genes associated with AD in humans and their orthologues in *C. elegans* according to the recently published Ortholist2 [12].

Likewise, a recent study has applied a systems biology approach, using data from transgenic models of AD in *C. elegans* that synthesize the Aβ protein, to find early molecular responses and genes and proteins important in the response to Aβ. In this way they have detected orthologous hub genes and transcription factors in worm, mouse and human, suggesting that Aβ expression affects similar pathways in these evolutionarily distant species, and therefore that *C. elegans* is a useful model to study the molecular processes involved in AD development [14].

However, although most AD-related genes possess orthologues in *C. elegans*, in some cases they do not, and this may represent an important limitation. Among the AD-related genes lacking orthologues in *C. elegans*, β-secretase (*BACE1*) is worth noting. Although *C. elegans* has a functional γ-secretase complex [15,16], the absence of β-secretase prevents the cleavage necessary to endogenously produce the β-amyloid peptide in *C. elegans*. Thus, in the nematode, APL-1 is first cleaved by α-secretase, releasing sAPL-1, and then by the γ-secretase complex [15,16]. In addition, the orthologue of *APP* in *C. elegans*, *apl-1*, does not possess the sequence of the β-amyloid peptide, as is the case with the redundant human amyloid β precursor-like proteins 1 and 2 (APLP1 and APLP2). For both reasons, *C. elegans* is unable to endogenously produce the β-amyloid peptide, and as we will see later, models of accumulation of this peptide have been constructed by exogenous expression methods. Another LOAD-related gene that lacks an orthologue in *C. elegans* is APOE, the main modifier of the sporadic form of the disease. Here, again, humanized models have been constructed by the exogenous expression of human genes.

## 4. The *C. elegans* Model: Advantages and Limitations

The *C. elegans* nematode model has numerous advantages for studying the mechanisms of human neurodegenerative diseases. First and foremost, the nematode has a relatively short generation and life cycle, around 3 weeks, together with low maintenance and propagation costs. *C. elegans* has a small nervous system, composed of only 302 neurons that form an invariant neuronal network, allowing processes and mechanisms to be traced at the level of individual neurons. In addition, its transparent body allows the visualization of all cell types at all stages of its life. This fact, together with the possibility of expressing fluorescent proteins in specific cells and tissues, is exceptionally useful for in vivo investigation of neuronal death or protein aggregation processes throughout the life of the worm. For example, *C. elegans* expressing Aβ1-42–GFP fusion proteins have allowed us to visualize the dynamics and aggregation of Aβ in vivo [17].

Another important advantage is the degree of anatomical, genetic and metabolic knowledge we have about the worm. We have a comprehensive characterization of the cell fate lineage and a complete map of neuronal connectivity [18,19]. We also have the complete genome sequence and very powerful genetic manipulation tools that allow us, for example, to generate mutant lines of any gene, to express humanized genes in specific cells or tissues, or to specifically silence any gene in adult worms by RNAi techniques. We have RNAi libraries with almost 20,000 different RNAi able to silence almost any gene in this model [20]. Numerous methods have also been developed for the functional characterization of neurodegeneration mechanisms in *C. elegans* [21]. All these genetic and functional tools have been used to build predictive models of human neurological diseases, including Alzheimer’s disease, based primarily on the significant homology between human and *C. elegans* genes (Table 1). Moreover, a number of precise techniques for the measurement of protein aggregation and rate of proteotoxicity have been developed specifically for this model [22]. Similarly, locomotion speed tracking analysis techniques have been developed that allow the accurate determination of motility disturbances and their recovery in multiple worms [23]. Thus, *C. elegans* offers an exceptional platform to investigate the cellular and molecular mechanisms of AD.

As mentioned above, most of the proteins involved in AD possess orthologues in *C. elegans*. Recently, protein interaction networks involved in AD that are conserved in *Homo sapiens* and *C. elegans* have also been identified. Interaction networks were assembled for the APP and tau proteins in *H. sapiens*, and their orthologues APL-1 and PTL-1 in *C. elegans*. Using Global Network Alignment for comparison, it was found that both APP processing and tau phosphorylation pathways are highly conserved in both organisms [24]. Therefore, the interaction pathways of these AD-related proteins are closely matched.

On the other hand, a unified platform of genome-scale metabolic models (GEMs) covering five large model animal models, including mouse (*Mus musculus*), rat (*Rattus norvegicus*), zebrafish (*Danio rerio*), fruit fly (*Drosophila melanogaster*), and worm (*Caenorhabditis elegans*), has recently been introduced. All GEMs can be consulted interactively through the Metabolic Atlas web portal [25]. We thus have a very comprehensive coverage of metabolic networks in *C. elegans* by considering both orthologous gene-based pathways and species-specific reactions.

Despite all its advantages, there are also important limitations to neurodegenerative disease research in *C. elegans*. On the one hand, we have the absence of some proteins important for disease pathogenesis, as mentioned above. However, in addition, it is clear that many mammalian features (e.g., a circulatory system, or the presence of myelinated neurons) do not exist in *C. elegans*, making it impossible to model these aspects of human physiology in the worm. Additionally, synaptic connectivity in nematodes is different from that in the human brain. Nematodes have no hippocampus or cortex, and the neural circuits are very different. Worms lack an adaptive immune system, but have an innate immune system to defend itself against pathogens [26]. They also have glial cells in contact with neurons [27]. Whether this is enough to model neuroinflammation is difficult to say. Clearly, invertebrates cannot be expected to perfectly model all aspects of human disease. Instead, basic responses to cellular insults appear to be quite conserved, such that the study of toxicity mechanisms in *C. elegans* may provide very useful information about human diseases. Table 2 summarizes the main pros and cons of using *C. elegans* models to study pathological processes in AD.

## 5. *C. elegans* Models to Study the Mechanism of Toxicity of Aβ

*C. elegans* is unable to generate β-amyloid peptides, due to the absence of β-secretase and the different amino acid sequence of this peptide in the APL-1 protein with respect to human APP. To circumvent this problem, in 1995 Christopher Link expressed a construct that directly produced human β-amyloid peptide in body wall muscle cells. The transgenic line obtained, CL2006, showed a clear phenotype: a progressive paralysis, usually starting in young adulthood, and premature death [28]. Immunohistochemical analysis further demonstrated that these animals accumulated extensive muscle-associated deposits that reacted with anti-Aβ antibodies. It has subsequently been shown that this line does not actually express the full Aβ1-42 peptide, but a truncated product lacking the first two N-terminal amino acids, Aβ3-42, which also aggregates forming fibrillary structures [29]. From there, a new line, called GMC101, has been developed that expresses full Aβ1-42 in muscle cells [30], generates faster paralysis than that induced by the expression of Aβ3-42, and has been widely used to study Aβ aggregation.

Link’s group also developed other lines with Aβ expression in muscle under different conditions [31,32]. For example, to allow for Aβ expression at a given time point in adulthood, line CL4176 has temperature-restricted Aβ expression. When worms are subjected to the permissive temperature, Aβ levels increase rapidly and paralysis occurs 24 h after the temperature increase. In addition, this same group also generated models with pan-neuronal expression of Aβ [31]. In these models, the worms did not show a phenotype with clear movement alterations, but intraneuronal deposits of Aβ and several alterations in behavioral assays demonstrating cognitive impairment could be observed. Specifically, these worms showed deficits in odor-associative learning behavior, serotonin-controlled behaviors, experience-dependent learning and egg laying [33].

### 5.1. Mechanism of Toxicity of Aβ Oligomers

Models expressing Aβ in muscle have been very useful to study the mechanism of Aβ toxicity, and have also been used to study another disease, inclusion body myositis (IBM), in which Aβ accumulation occurs precisely in muscle and probably shares many pathogenic mechanisms with AD. On this basis, these models have been used to study the toxic effects of Aβ in muscle and it has been seen, for example, that copper treatment increases Aβ aggregates and decreases oligomers, which improves motility and synaptic function, and delays paralysis [34]. In fact, it had already been shown in the strain CL2006 that large Aβ aggregates do not correlate with toxicity, whereas there is correlation between toxicity and the presence of small oligomers close to muscle fibers [35].

This is a topic that has been extensively studied in AD patients, in whom cognitive impairment does not correlate with the accumulation of amyloid plaques, but rather with the presence of toxic soluble oligomers. Recent work explained this fact by showing that Aβ oligomers adopt a non-standard secondary structure called “α-sheet”. These oligomers form in the initial phase of aggregation, when Aβ-associated cytotoxicity is maximal, before eventually forming non-toxic folded β-sheet fibrils [36]. In the same work, peptides adopting the same α-sheet structure were designed to be able to bind strongly to toxic oligomers, inhibiting in vitro aggregation and neurotoxicity in neuroblastoma cells. In addition, these synthetic peptides also inhibited paralysis in a model of Aβ overexpression in *C. elegans*, so they might be useful in the treatment of AD and other amyloid diseases [36].

Since the toxic species are small oligomers and not amyloid aggregates, it is essential to understand the early stages of nucleation of amyloid peptides to form oligomers. In this regard, it was recently seen in humans and *C. elegans* that there is a small, disordered and highly conserved protein called SERF (human)/MOAG-4 (*C. elegans*), which has a strong ability to accelerate the primary nucleation of aggregator-prone proteins such as Aβ or α-synuclein [37]. This process appears to be mediated by SERF/MOAG-4 interactions with negatively charged segments in aggregation-prone proteins [38]. These charge interactions between cellular modifiers and amyloidogenic proteins could be new therapeutic targets to block the generation of toxic oligomers.

Several interesting models have recently been described in *C. elegans* for the study of oligomer formation and its effects. One of them is an optogenetic model based on the expression in *C. elegans* of Aβ with a fluorescent marker that rapidly oligomerizes in the presence of blue light. Metabolic defects, loss of mitochondria and inflammation only occurred after light-induced Aβ oligomerization, and Aβ expression alone was not sufficient for this [39]. Another important technique for studying the oligomerization process was the development of oligomer-specific antibodies, allowing the precise quantification of oligomers, both in vitro and in the *C. elegans* GMC101 model [40]. Another model to study Aβ aggregation involves the coexpression of fluorescently labeled or unlabeled Aβ1-42, either under control of a pan-neuronal promoter or under control of a body wall muscle-specific promoter, in order to allow adequate Aβ tracking [41]. Aβ expression in this model caused severe physiological defects, neuronal dysfunction and neurodegeneration. Fluorescence lifetime imaging microscopy (FLIM) allowed a quantification of amyloid fibril formation with aging that showed that Aβ aggregation begins in a subset of anterior head ganglion neurons, the six IL2 neurons. Moreover, RNAi depletion of Aβ in these IL2 neurons globally delayed Aβ aggregation and pathology. These neurons could represent a site of initiation of amyloid propagation [41].

The length of the Aβ peptide is also important in determining its toxic effects. It was already known that Aβ1-42 is more toxic than Aβ3-42, and this fact had also been seen in *C. elegans* comparing strains CL2006 and GMC101 [29,30]. More recently, it has been described that Aβ1-38 is able to mitigate the phenotype induced by Aβ1-42 expression in the GMC101 strain [42]. These results suggest that within the pool of Aβ variants, it is possible that some are neuroprotective, which may be important in order to understand the failure of many therapies indiscriminately targeting the Aβ peptide.

### 5.2. Aβ Toxicity and Insulin Signaling

The CL2006 strain was used to demonstrate that Aβ toxicity was reduced in *C. elegans* when aging was slowed by decreased signaling through the insulin/insulin growth factor-1-like (IIS) pathway, providing a link between the aging process, insulin pathway signaling and Aβ aggregation-mediated proteotoxicity [35]. Furthermore, it could be shown that protection against Aβ toxicity is independent of the increase in longevity induced by IIS reduction. In young adults (day 1–5), daf-2 RNAi knockdown of IIS pathway activity produced both effects, but later, at day 9, IIS knockdown only protected against Aβ toxicity, but did not increase longevity. The protective effect against Aβ was mediated by two transcription factors, heat shock factor 1 (HSF-1) and DAF-16 (FOXO orthologue), and the mechanism appears to be that IIS depletion facilitates the formation of large aggregates, less toxic than oligomers [43,44]. The protective effect of IIS reduction was also confirmed in a murine model [43].

Antidiabetics such as metformin delayed paralysis linked to Aβ expression and enhanced neurotransmission by acetylcholine in a *C. elegans* model expressing Aβ in muscle [45]. This work supports the idea that antidiabetic drugs and metabolic modulators may be effective against AD. Likewise, in a *C. elegans* model expressing Aβ at a low level in neurons (GRU102), metabolic alterations in the Krebs cycle were identified that appeared before any increase in protein aggregation. Additionally, here, metformin treatment reversed the Aβ-induced metabolic defects, reduced protein aggregation, and normalized the longevity of the GRU102 strain [46]. Therefore, metabolic dysfunction appears to be an early event and origin of Aβ-induced pathology and thus a promising target for therapeutic intervention.

The fact that some drugs developed to treat diabetes also have a positive effect on AD patients suggests to some authors that there may be a common metabolic basis for both diseases. There are also other common findings, such as the formation of misfolded proteins, advanced glycation end products (AGEs), mitochondrial disorders, the generation of oxidative stress, insulin resistance and abnormal glucose metabolism [47].

### 5.3. Aβ Toxicity and Proteostasis

Altered proteostasis plays an essential role in AD and other neurodegenerative diseases. The function of the proteostasis machinery is to maintain a correctly folded proteome. However, aging may lead to the expression of aggregation-prone proteins or to the accumulation of damaged proteins. This may disrupt the proteostasis machinery, with the subsequent accumulation of misfolded proteins. Results obtained in *C. elegans* models have been very useful to understand the mechanisms of this process (see [48,49]).

Inactivation of the *epi-1* gene, an α-chain of laminin, resulted in increased proteotoxicity and significantly accelerated the onset of paralysis in worms overexpressing Aβ (CL2006). Interestingly, reduced signaling through the IIS pathway was able to rescue paralysis caused by *epi-1* silencing [50].

Treatment of AD models in *C. elegans* with a proteasome activator reduced paralysis and Aβ deposition, suggesting that the proteasome may be a therapeutic target in AD [51]. Similarly, misfolded proteins and Aβ have been shown to activate the transcription factor NRF1/SKN-1A, which directs the expression of proteasome subunits. In an AD model in *C. elegans*, increasing NRF1/SKN-1A slowed Aβ accumulation and cellular dysfunction [52]. These data suggest that NRF1/SKN-1A monitors cellular protein folding and adjusts proteasome capacity to meet the demands of protein quality control pathways. This regulatory axis is critical for healthy aging and may perhaps be a target for the treatment of age-related diseases.

A study using strain GMC101 presented evidence that changes in protein aggregation that occur during middle age are very important for initiating Aβ aggregation, and that it is the highly insoluble proteins of aged *C. elegans* GMC101, but not those of young individuals, that are capable of initiating Aβ aggregation in vitro [53]. Widespread misfolding and aggregation of proteins with age could be critical for the onset of pathogenesis, and therefore should be one of the targets of therapeutic strategies in neurodegenerative diseases. Another study in this same model showed that induction of the mitochondrial stress response is essential for the maintenance of mitochondrial proteostasis. The activation of mitochondrial proteostasis increased the fitness and longevity of GMC101 worms and reduced Aβ aggregation. Activating mitochondrial proteostasis is therefore a new option to delay proteotoxicity diseases, such as AD [54].

### 5.4. Toxicity of Aβ and Gene Expression

These *C. elegans* models have also been used to search for genes whose overexpression or silencing reduces Aβ accumulation and paralysis. In one such study, the arsenite-inducible protein AIP-1 was identified for its ability to reduce Aβ accumulation and attenuate paralysis. In addition, *AIRAPL*, the human homolog of *aip-1*, produced the same effect when expressed in the CL4176 strain expressing Aβ in body wall muscle in a temperature-restricted way [55]. Similarly, deletion of the phospholipase D gene (*pld-1*) in an AD model overexpressing Aβ markedly improved the motor phenotype and worm size, supporting the idea that phospholipase D plays an important role in neurodegeneration [56]. Similarly, in a screening of CL4176 model mutants resistant to Aβ toxicity, the gene *hmgs-1* (orthologue of human HMG-CoA synthase), whose silencing delayed worm paralysis, was discovered [57]. It has also been described that the activation of *PPARα*/*nhr-49* (nuclear hormone receptor involved in lipid signaling) inhibits the steroid signaling pathway, which increases the nuclear translocation of DAF-16 and inhibits the Aβ-induced phenotype in an AD model in *C. elegans* [58]. Finally, deletion of the *dld-1* gene, which encodes for dihydrolipoamide dehydrogenase, has also been shown to have a protective effect in *C. elegans* models overexpressing Aβ, probably through reduced energy metabolism and proteasome activation [59,60]. This effect is consistent with the fact that variants of the human orthologue of this enzyme are linked to LOAD.

*C. elegans* models of Aβ overexpression have been used to validate the effect of novel LOAD-associated genes found in human GWAS studies by RNAi silencing studies of orthologues in *C. elegans*. In this way, a functional role of several of these genes has been demonstrated [61]. The effect in *C. elegans* of RNAi silencing of genes orthologous to human genes differentially expressed with age has also been studied, finding a large number of genes whose silencing increased longevity and in some cases also rescued the phenotype of AD models [62]. Most of those genes had not been previously described for that function. These data validate *C. elegans* as a tool to find new human genes with effects on aging and AD.

A new model in *C. elegans* of AD with neuronal overexpression of Aβ1-42 together with pro-aggregating tau or anti-aggregating tau was recently described. The model showed decreased longevity, deficits in associative learning, increased protein aggregation and neuronal loss. Global RNA-seq transcriptomic analysis showed that eight of the regulated genes in *C. elegans* matched the top 60 human AD-related genes, validating the use of the model to search for genes related to longevity and AD [63]. Comparing the effect of overexpression of only Aβ1-42, only wild-type tau or both, it could be seen that there was a synergistic effect of co-expression of both peptides in the genesis of neuronal dysfunction [64].

Preincubation with the chaperone clusterin, one of the proteins that pose a genetic risk for LOAD, antagonized the toxic effects of *C. elegans* treatment with Aβ oligomers [65]. These data corroborate the interaction of clusterin with biologically active regions of oligomers, providing a molecular basis for the neuroprotective effects of the chaperone. Similarly, monomers of non-phosphorylated tau were also effective in antagonizing the toxic effects of Aβ oligomers [66]. These monomers could therefore have a neuroprotective effect.

The alterations in gene expression induced by Aβ expression in *C. elegans* have also been investigated, and how they compare with those induced by a synthetic aggregating protein. In this way, specific Aβ-induced and non-specific alterations were identified, with the former including several genes related to accelerated aging, proteasome function and mitochondrial function [67].

In another study, 515 human proteins with aggregating capacity were investigated, of which 56 were more abundant in aged muscle and 21 less abundant. The orthologues of seven of these proteins were silenced in *C. elegans* by RNAi, and six of the seven treatments decreased protein aggregation in aged nematodes, and rescued motility in the CL4176 model. These proteins are potential targets for sarcopenia and AD treatments [68].

A genome-wide RNA-interference-based screening of genes regulating behavioral impairment in *C. elegans* found two epigenetic regulators, *baz-2* and *set-6*, that accelerate behavioral impairment in *C. elegans* by reducing mitochondrial function, repressing the expression of mitochondrial proteins encoded by the nucleus. This finding is relevant to AD, given that expression of the human orthologues of these *C. elegans* regulators, *BAZ2B* and *EHMT1*, in the frontal cortex increases with age and correlates positively with Alzheimer’s disease progression [69].

### 5.5. Aβ Toxicity and Oxidative Stress

There are numerous studies performed in the *C. elegans* model that link oxidative stress and AD. There is, for example, evidence that the oxidation of K^+^ channels by ROS increases the activity of these channels and reduces neuronal excitability, a phenomenon that occurs during aging and may be a general phenomenon in many neurodegenerative diseases [70].

More specifically, the CL2006 strain model has been used to demonstrate that Aβ accumulation leads to increased iron content and ROS production, suggesting that alterations in iron homeostasis could be involved in the pathogenesis of AD [71]. Similarly, the suppression of mitoferrin-1 by RNAi prolonged the lifespan of strains CL2006 and GMC101 and reduced the rate of paralysis in strain GMC101, probably through a reduction in mitochondrial iron content and mitochondrial ROS level in these strains. Therefore, the knockdown of mitoferrin-1 might contribute to slowing the progression of AD [72].

The protective effect of the mitochondrial thioredoxin TRXR-2 has also been described in the CL2006 model [73], also suggesting the importance of mitochondrial redox homeostasis in the pathogenesis of AD. Moreover, another thioredoxin present in the endoplasmic reticulum, DNJ-27 (orthologue of human ERdj5), also has a protective effect against Aβ toxicity and its silencing potentiates motor impairment and paralysis in the CL2006 model [74]. Alterations in mitochondrial dynamics and cytoplasmic proteostasis also participate in the protective mechanism of DNJ-27.

The importance of oxidative stress is also highlighted by the fact that the transcription factor SPR-4 protects against oxidative stress and Aβ toxicity in a *C. elegans* model of Aβ overexpression in glutamatergic neurons. SPR-4 is the orthologue of REST, a transcription factor that reduces its expression in AD and in normal individuals represses genes that promote cell death and AD, and induces stress response genes [75]. Consistently, loss-of-function mutations in the *C. elegans* REST orthologous genes *spr-3* and *spr-4* increased neural excitability and reduced the longevity of long-lived daf-2 mutants [76].

The endogenous inhibitor of NO synthase asymmetric dimethylarginine (ADMA) is upregulated in the CL2006 model, and ADMA treatment of this strain exacerbated Aβ-induced paralysis and oxidative stress, suggesting that ADMA may participate in the pathogenesis of AD [77].

### 5.6. Other Models of AD with Aβ Overexpression in C. elegans

A mutation in the APP protein (A673V) corresponding to position 2 of the Aβ peptides was found to cause an early-onset AD-type dementia in a homozygous individual [78]. A new model of AD in *C. elegans* was obtained by expressing wild-type or mutated Aβ1-40 (Aβ1-40A2V) in neurons. The expression of both WT and A2V Aβ1-40 reduced nematode longevity, causing behavioral defects and neurotransmission alterations that were worse in A2V worms. In addition, Aβ1-40A2V had a greater tendency than Aβ1-40WT to form soluble oligomeric species in vivo that accumulate within neuronal cells [79].

Another model of AD in *C. elegans* was obtained by the constitutive pan-neuronal expression of Aβ1-42 at different doses, and selecting a line with a late-onset behavioral disturbance phenotype. The model showed age-dependent neuromuscular and behavioral defects, together with an early ATP deficit that was followed by alterations in complexes I and IV of the mitochondrial respiratory chain. Similar changes have been described in human AD pathogenesis. These results suggest an important role for mitochondrial metabolic dysfunction in AD [80].

A minimal damage model was also generated by expressing Aβ1-42 from a single-copy insertion of the gene in a single pair of glutamatergic sensory neurons, the BAG neurons. This model showed subtle changes in CO_2_ response and Ca^2+^ dynamics together with behavioral changes in speed and direction of travel, but these were not sufficient to generate neurotoxicity [81].

Since *C. elegans* does not possess orthologues of ApoE, one of the major modulatory genes in human AD, the models that have been generated in *C. elegans* to study the effect of different ApoE alleles have been based on coexpression in the neurons of Aβ and the different human ApoE alleles, ApoEε2, ApoEε3 and ApoEε4 [82]. Coexpression of human APOEε2 with Aβ alleviated the neurodegeneration. In contrast, coexpression of the APOEε4 allele did not modify neurodegeneration, suggesting that APOEε2 is neuroprotective but that APOEε4 lost this property. The APOEε3 allele showed an intermediate phenotype. This model is very interesting to explore the effects of ApoE alleles on Aβ-induced neurotoxicity.

## 6. *C. elegans* Models to Study the Function of APP

The *C. elegans* homologue of the APP protein, called APL-1, is structurally similar to its mammalian homologue and shares sequence homology in the N-terminal domains and in the C-terminal intracellular domain, which is the most conserved. However, APL-1 does not contain the sequence of the amyloid-β peptide, as is also the case with the functionally redundant mammalian APP homologs, APLP1 and APLP2. Although APP is a central protein in the pathogenesis of AD and numerous mutations in this protein are responsible for EOAD, the physiological function of this protein is still poorly understood. The *C. elegans* model has been used extensively to gain insight into its function, based on manipulation of the orthologous gene, *apl-1*.

The first data were obtained by suppressing or overexpressing *apl-1*. Loss of *apl-1* is lethal to larvae because it disrupts molting and morphogenesis, but can be rescued by neuronal expression of the extracellular domain of APL-1 (sAPL-1, which is normally formed by the action of α-secretase). Overexpression of APL-1 also caused several defects, including larval lethality, and this effect could be partially rescued by decreasing the activity of *sel-12*, the orthologue of human presenilin. APL-1 is thus a multifunctional protein involved in molting, reproduction, locomotion and morphogenesis. Moreover, its extracellular domain (sAPL-1) is particularly important, able to act noncell-autonomously and may have essential functions in the nervous system [15]. Loss of *apl-1* also independently generated defects in cholinergic neurotransmission at the synaptic level, which could also be rescued by sAPL-1 [83].

It was later shown that APL-1 signaling was dependent on the activity of the transcription factor DAF-16 (orthologue of FOXO) and the nuclear hormone receptor DAF-12, and modulated metabolic pathways responsible for development, body size and egg laying [84]. Likewise, pan-neuronal expression of APL-1 disrupted olfactory and gustatory learning behaviors and habituation to touch, also through a reduction in insulin pathway activity [85]. The proposed mechanism for this effect would be that after cleavage of APL-1, the released product (sAPL-1) would be able to reduce insulin signaling to activate DAF-16 and/or DAF-12, thus affecting development, various metabolic functions, and associative and non-associative learning pathways. The effect of the sAPL-1 fragment could be mediated by competition with insulin-like peptides (ILPs) for binding to the insulin/IGF-1 receptor (DAF-2). Alternatively, it could be acting on a receptor protein tyrosine phosphatase (RPTP) able to modify downstream signaling of the insulin/IGF-1 receptor [16,86].

Although overexpression of APL-1 in neurons reduces longevity, ubiquitous overexpression produced an increase in longevity. In fact, overexpressing APL-1 in cells of the hypodermis was sufficient to produce this increase in longevity, and overexpressing the sAPL-1 fragment in them was also sufficient. Increased longevity also required signaling through the transcription factor FOXO/DAF-16, the heat shock factor HSF-1, and the DAF-12 receptor [87]. Taken together, these results suggest that *apl-1* expression in neurons is important for viability, while in hypodermal cells it modulates longevity.

By studying the interactome of the intracellular domain of APP, it has been shown that APP binds to the PIKfyve complex. This is a protein kinase that participates in the synthesis of phosphatidylinositol-3,5-bisphosphate (PIP_2_) in the endosomal compartment. Endosomal PIP_2_ is essential for endosomal sorting, morphology and function, including Ca^2+^ permeability. Loss of PIKfyve function induces neurodegeneration in mammals, and therefore its regulation by APP may be important for AD pathogenesis [88]. A role for APL-1 in axon guidance and neural circuit formation (neural circuit) has also been described, such that the inactivation of APL-1 results in axon misdirection (axon mistargeting) [89].

It is also interesting to mention that, in a model of human APP overexpression in *C. elegans*, APOE4, but not APOE3, was able to cooperate with APP to accelerate and amplify the pattern of cholinergic neurodegeneration caused by APP. This model may be very useful in determining the molecular mechanisms underlying the synergy between APP and APOE4 to induce neurodegeneration [90].

## 7. *C. elegans* Models to Study the Function of Presenilins

The *C. elegans* genome contains three genes orthologous to human presenilins [91]. One of them, *spe-4*, is only expressed in spermatozoa during spermatogenesis and has no relevance for AD models. The other two genes are *sel-12* (suppressor/enhancer of lin-12) and *hop-1* (homolog of presenilin). Both are somatically expressed, have similar functions and can substitute for each other under conditions that allow the de-repression of *hop-1*. However, while *hop-1* null mutants have no apparent phenotype, all known mutations in *sel-12* have significant alterations in egg laying, apart from additional deficits.

Models of AD in *C. elegans* based on presenilin mutations are therefore based on *sel-12* mutations. Moreover, these models are characterized by the fact that they have allowed the study of the role of presenilins in Ca^2+^ homeostasis. Specifically, in *sel-12* mutants, increased Ca^2+^ transfer from the endoplasmic reticulum (ER) to the mitochondria has been observed, producing an increase in mitochondrial Ca^2+^ content that stimulates mitochondrial respiration and increases mitochondrial superoxide production. Mitochondrial metabolic defects and neurodegeneration in *sel-12* mutants are prevented by reducing ER Ca^2+^ release, mitochondrial Ca^2+^ uptake or mitochondrial superoxide production. Therefore, alterations in ER and mitochondrial Ca^2+^ homeostasis are essential to explain the defects observed in the mutants, and allow the generation of alternative therapeutic targets for the treatment of neurodegenerative diseases [92,93,94].

*Sel-12* mutants also have widespread defects in proteostasis, which are prevented by reducing Ca^2+^ transfer from the ER to the mitochondria or by treatment with antioxidants. These data suggest that altered Ca^2+^ homeostasis between ER and mitochondria in *sel-12* mutants generates alterations in proteostasis due to increased oxidative stress [95]. In addition, mutations in *sel-12* result in the hyperactivation of the mTORC1 pathway, due to increased mitochondrial Ca^2+^ and consequent mitochondrial activation and increased energy production. This hyperactivation of mTORC1 potentiates defects in proteostasis and neurodegeneration, and blockade of mTORC1 allows for partial reversal of the proteostasis defects and neurodegenerative phenotypes of *sel-12* mutants [96].

The role of Ca^2+^ homeostasis in AD has also been studied in relation to the association between the P86L polymorphism of calcium homeostasis modulator 1 (CALHM1) and AD risk [97]. The mutation results in decreased Ca^2+^ permeability through these plasma membrane Ca^2+^ channels [98]. Therefore, a reduced Ca^2+^ permeability through this channel is linked to an increased risk of AD. *C. elegans* has an orthologue of this channel, called *clhm-1*, which is also a voltage- and extracellular Ca^2+^-regulated Ca^2+^-permeable ion channel. Loss of *clhm-1* in body wall muscle impairs locomotion and motility defects can be rescued by muscle-specific expression of *clhm-1* or human CALHM1. Overexpression of *clhm-1* is toxic, causing degeneration through a necrotic-like mechanism that is partially dependent on Ca^2+^ influx. Taken together, altered neuronal Ca^2+^ homeostasis caused by inappropriate functioning of this channel could favor the onset of neurodegenerative diseases such as AD [99].

## 8. *C. elegans* Models to Study the Function and Mechanism of Toxicity of Tau

Tau protein belongs to the family of microtubule-associated proteins (MAPs). MAPs act together with tubulin heterodimers to assemble microtubules. In mammals there are three types, according to their size: MAP1 (>250 kDa), MAP2 (200 kDa) and MAPT (tau, 50–70 kDa). MAP2 and tau are co-expressed in most neurons, although MAP2 is mainly found in dendrites and tau is concentrated in axons. Tau has a prominent role in microtubule stability, but when it is excessively phosphorylated it detaches from microtubules and forms the neurofibrillary tangles (NFTs) characteristic of AD and many other diseases collectively referred to as tauopathies. Mutations in the MAPT gene have been described, leading to frontotemporal dementia (FTD) or other tauopathies, and some mutations also act as risk factors for AD [100].

Regarding models of tauopathies in *C. elegans* [101], most models have been generated by the pan-neuronal expression of normal or mutated human tau, although models with tau expression in selected neurons, models with tau expression in other tissues, and mutants of the endogenous tau orthologous protein, *ptl-1*, have also been used.

### 8.1. Models of Tau Expression in the Nervous System of C. elegans

The first models were performed by the pan-neuronal expression of normal human tau and tau with the mutations associated with frontotemporal dementia (FTD) [102]. The pan-neuronal expression of tau produced progressive uncoordination in locomotion, with accumulation of insoluble tau and phosphorylation of tau at many of the sites where it is commonly phosphorylated in FTD, AD and other tauopathies. Tau-expressing *C. elegans* lines with FTD mutations had a more severe uncoordinated phenotype and greater neurodegeneration than those with normal tau expression [102]. Subsequently, the same group performed a genome-wide RNAi screen in this model looking for genes capable of modifying the phenotype induced by tau expression [103]. In this study they found 75 genes whose silencing enhanced the uncoordinated phenotype induced by mutant tau expression. The genes identified included kinases, chaperones, proteases, phosphatases, enzymes and other genes of unknown function.

This same model was then used to identify genes involved in tau neurotoxicity from a genetic screen to look for mutations that enhance the tau-induced uncoordinated phenotype. These studies identified two genes, *sut-1* and *sut-2*, whose mutations lead to an improvement in the phenotype [104,105]. Furthermore, overexpression of *sut-2* increased tau-induced neuronal dysfunction, neurotoxicity and insoluble tau accumulation in *C. elegans* [106]. Interestingly, the human orthologue of *sut-2*, *MSUT2*, also undergoes modifications in AD patients and its silencing in tau-overexpressing cell cultures reduces tau aggregation [106]. The data from the *C. elegans* model are therefore extrapolable to AD.

Regarding its function, *sut-2*/*MSUT2* is a poly(A) deadenylase, but it is interesting to note that not all poly(A) deadenylases produce the same effect. Loss-of-function mutations in the *ccr-4* and *panl-2* genes of *C. elegans* increased the tauopathy phenotype, but loss of *parn-2* reduced it. The enhanced tauopathy phenotype induced by loss of *ccr-4* was alleviated by the loss of *sut-2* and, contrarily, overexpression of *sut-2* aggravated it even in the absence of *parn-2*. Thus, there is not a simple relationship among the suppression of tauopathy and extended poly(A) tails. Instead, there seems to be a more complex relationship between tau, *sut-2*/*MSUT2* function, and RNA metabolism [107].

Another model of tauopathy in *C. elegans* was obtained by expressing normal or mutant tau (P301L and R406W) in mechanosensory touch neurons. Worms expressing normal tau showed a small reduction in the response to touch, while worms expressing mutant tau showed a much larger and progressive effect. Moreover, accumulations of tau in the cell body and neuronal processes were observed in many of the neurons undergoing degeneration [108].

Fatouros et al. [109] created another model of tauopathy by expressing pro- or anti-aggregating tau species in the nervous system of *C. elegans*. Animals expressing the aggregating tau species showed greater impairment of motility and obvious neuronal dysfunction. Those expressing the anti-aggregating combination had a rather mild phenotype. The model also allowed the testing of tau aggregation inhibitory compounds, some of which were shown to be effective in improving motility and delaying the accumulation of neuronal defects [109]. Recently, it was shown that the anti-aggregating fragment is able to inhibit tau aggregation in vitro and in a *C. elegans* model of tau aggregation, it suppresses tau-mediated neuronal dysfunction and ameliorates alterations in locomotion [110].

Another model was obtained by generating lines that overexpressed human MAP2 or tau proteins pan-neuronally. Both are proteins with high homology, but only tau, and not MAP2, is part of the neurofibrillary tangles in AD patients. However, in *C. elegans* both tau and MAP2 induced neurotoxicity, but without forming aggregates in either case. This suggests that the involvement of MAP2 in AD pathogenesis cannot be excluded by the absence of aggregates alone [111].

To investigate the mechanism of neurodegeneration generated by the A152T tau mutation, which appears in several tauopathies, *C. elegans* strains expressing normal or mutant tau at the neuronal level have been generated. While the expression of normal human tau induces mild locomotion incoordination, mutant A152T tau protein induces severe paralysis accompanied by acute neuronal dysfunction. As in other models already mentioned, tau mutant worms did not accumulate insoluble tau aggregates. Instead, only soluble oligomeric tau was detected [112]. The mechanism of neurodegeneration is a necrosis of glutamatergic neurons due to a sum of factors, including alterations of the adenylate cyclase pathway, excitotoxicity, and dysregulation of intracellular Ca^2+^ with increased Ca^2+^ release from the ER [113].

The role of the relative ratio between tau and tubulin levels was tested by generating a series of *C. elegans* transgenic lines with pan-neuronal expression of tau at various expression levels, or alternatively with reduced tubulin levels. In all cases, increasing the tau/tubulin ratio promoted increased toxicity, suggesting that the ratio may be determinant for tau toxicity [114].

The effects of tau on mitochondrial function were studied in *C. elegans* models overexpressing normal or mutated human tau (hP301L). In the nervous system of *C. elegans*, expression of normal tau reduced mitophagy, whereas expression of hP301L tau completely inhibited it. Tau specifically blocked Parkin recruitment to defective mitochondria by sequestering it in the cytosol [115]. Similarly, in both Aβ and tau expression models, stimulation of mitophagy (by NAD+, urolithin A or actinonin supplementation) reversed memory defects. Thus, defects in mitophagy appear to be a pivotal event in AD pathogenesis and modulators of mitophagy could be therapeutically important [116]. The same conclusion can be drawn from a new model of single-copy expression of tau in tact receptor neurons. This model lacked a basal phenotype, but the presence of certain phosphorylation-simulating mutations facilitated the progressive loss of tact, the appearance of morphological abnormalities, and the loss of mitophagy in response to mitochondrial stress [117].

### 8.2. Effects of Tau Phosphorylation in C. elegans

In another model of human tau expression, *C. elegans* strains were generated with pan-neuronal expression of human tau and a pseudo-hyperphosphorylated form of tau (PHP tau), with glutamate-substituted serine/threonine residues at positions that are commonly phosphorylated in AD. In these models they were also able to observe that human tau is abundantly phosphorylated in *C. elegans* and exhibits conformational changes similar to PHP tau and human tau forming paired helical filaments (PHF). Both the wild-type tau and PHP tau expression models induced the appearance with age of an uncoordinated locomotion phenotype in the absence of neuronal degeneration. However, only PHP tau expression induced a defective pattern of motor neuron development. Thus, although *C. elegans* is able to abundantly phosphorylate human tau to an AD-like state, only stable AD-like modification of tau was able to induce neuronal developmental defects [118]. This same model was later used to demonstrate that inhibition of mitochondrial dihydrolipoamide dehydrogenase by various mechanisms results in hyperglycemia and increased tau phosphorylation [119].

Tau is strongly phosphorylated by tau tubulin kinases 1 and 2 (TTBK1/2). A new model of *C. elegans* was developed that coexpresses tau/TTBK1 or tau/TTBK2, resulting in a synergistic exacerbation of the abnormalities. These data suggest a possible etiology for some tauopathies based on a mechanism of neurodegeneration driven by kinase activation [120].

### 8.3. Models with Tau Expression in Muscle Cells

Finally, a *C. elegans* model of tau expression in body wall muscle cells was recently developed. The goal was to develop a model of tauopathy in *C. elegans* that had clear pathological phenotypes in cells that could be modified by RNAi methods. The problem with previous models is that they all express tau in neurons, which do not respond to the usual RNAi methods. The expressed tau is phosphorylated at epitopes canonically associated with AD and the strain shows alterations in egg laying, growth rate, paralysis, thrashing frequency, crawling and lifespan. These defects are suppressed by RNAi directed against tau mRNA. This model could be a useful tool for applying RNAi screening to the study of mechanisms influencing human tau hyperphosphorylation and toxicity [121].

### 8.4. Models Using the Endogenous ptl-1 Gene

As mentioned above, some studies have also used the endogenous *ptl-1* gene to study the functional role of tau. *C. elegans* has only one homolog of the microtubule-associated proteins (MAPs), called PTL-1, which has been used to investigate the functional role of tau without the complication of having other functionally redundant proteins, as occurs in mammals with the rest of the MAPs. Mutants of *ptl-1* have altered microtubule-based axonal transport [122] and reduced longevity [123]. Decreased lifespan can be rescued by re-expression of PTL-1 in the *ptl-1* null mutant (but not by expression of human tau), but dosage is critical, because excess expression produces negative effects [123]. Moreover, rescue is cell autonomous, because re-expression of PTL-1 only in one neuronal type succeeds in rescuing only that type, and to rescue a lifespan decrease, it is necessary to re-express it pan-neuronally [124]. In any case, PTL-1 has a key role in neuronal maintenance with age and in the regulation of longevity, and its study may provide important clues about the physiological role of MAPs.

## 9. Use of *C. elegans* Models of Alzheimer’s Disease to Find New Drugs and Therapies

As we have seen so far, multiple models of toxicity induced primarily by the expression of normal or mutated Aβ or tau in *C. elegans* have been developed. These models have made it possible to test many potential pharmacological treatments for assessing the reversal of toxic effects. Numerous candidate AD modulators have thus been described [125]. In addition, new high-throughput drug screening techniques applied to *C. elegans* have recently been developed, representing a very powerful approach to identify new chemical modulators of these processes [126]. For example, an advanced computer vision system for characterizing *C. elegans* behavior has been described, which allows the identification of behavioral deficits and the simultaneous multiparametric monitoring of up to 5000 animals in Aβ42 expression models [127]. This system can facilitate high-throughput studies in the context of drug discovery.

The following is a list of compounds that have been shown to be effective in alleviating the phenotype in *C. elegans* AD models (see Table 3).

### 9.1. Antidiabetics

It has already been mentioned that reducing insulin/IGF signaling cascade (IIS) activity is beneficial with regard to the toxic effects of Aβ and other proaggregating proteins. This idea led to the testing of various antidiabetics to treat neurodegenerative diseases. We have already commented that metformin reversed Aβ-induced toxic effects in several *C. elegans* models expressing Aβ [45,46]. Similar effects have been obtained with the inhibitor NT219, which also protected nematodes from Aβ-associated proteotoxicity without affecting longevity [128].

The effect of a combination of metformin and lithium (aimed at improving proteostasis) has also been studied in a *C. elegans* model of pan-neuronal Aβ expression. Worms treated with this combination treatment enjoyed significantly compressed morbidity and increased longevity, even compared with untreated non-transgenic animals [129].

### 9.2. Antioxidants

Clioquinol, an 8-hydroxyquinoline, rescued a model of Aβ toxicity in *C. elegans* in which Aβ is expressed in glutamatergic neurons. Clioquinol promoted the degradation of Aβ oligomers within the secretory and endosomal compartments, indicating that it is possible to target toxic oligomers directly in these compartments [130]. The 8-hydroxyquinolines were developed for their ability to inhibit the metal-mediated generation of reactive oxygen species from Aβ:Cu complexes, but their mechanism of action is not known in detail and it has also been described that they are able to associate with Aβ directly to form a stable non-toxic dimer [131].

The mitochondria-targeted antioxidant MitoQ prolonged the lifespan and improved the health of a *C. elegans* model overexpressing Aβ [132]. The organoselenium compound diphenyldiselenide (PhSe)2, which is characterized by antioxidant and anti-inflammatory properties, was also shown to be effective in a transgenic *C. elegans* AD model of Aβ-induced toxicity [133].

### 9.3. Compounds with Anti-Amyloidogenic Mechanism by Direct Interaction with Oligomers

One of the most interesting lines of research into new treatments for AD is the search for molecules capable of blocking the production of Aß oligomers. Following this idea, much work has been carried out in the search for antibodies directed against Aß42. Aprile et al. [134] presented an “antibody scanning” strategy, which allowed them to identify two antibodies that specifically target the initial stages of nucleation, essential for the generation of the Aβ42 oligomers. These two antibodies reduce toxicity in a *C. elegans* model by acting through two mechanisms: delaying the start of aggregation and blocking the proliferation of the aggregates [134].

There are also small peptides capable of interacting with oligomers. The 24-residue peptide humanin and a synthetic derivative of it were found to be able to interfere with the formation and toxicity of Aβ oligomers in the CL4176 model [135]. Lactoferrin-derived peptides have also been described as inhibitors of prolyl endopeptidase and they also inhibit oligomer formation and reduce paralysis in a model of Aβ expression in *C. elegans* [136].

An in silico strategy was recently used to design a bicyclic peptide targeting the C-terminal region (residues 31–42) of the Aβ42 peptide. This peptide modified the Aβ42 aggregation process in such a way that it reduced its toxicity in a *C. elegans* model. These results illustrate the utility of this kind of approach to design specifically targeted peptides [137]. Following with the use of targeted peptides, circular disulfide-rich peptides from *Clitoria ternatea* (*C. ternatea*) delayed Aβ-induced paralysis in the worm strain CL4176 (with muscle Aβ overexpression), as well as Aβ-induced chemotaxis defects in the worm strain CL2355 (with neuronal Aβ overexpression). This effect is probably also mediated by the inhibition of Aβ oligomerization [138].

Chemical kinetics methods allow the effects of small molecules on specific microscopic steps of Aβ42 aggregation to be quantified with high precision. This approach has allowed the development of a strategy of drug discovery against Aβ42 aggregation based on changes in nucleation and elongation rate constants caused by candidate molecules. Thus, a set of compounds were identified and then tested in a *C. elegans* AD model. This strategy represents a powerful approach to systematically identify new molecules capable of inhibiting specific microscopic steps of the protein aggregation reaction [139].

In the search for optimal strategies to inhibit the formation of toxic oligomers, a strategy has also been developed to optimize the administration protocols of compounds capable of inhibiting aggregation processes. According to this strategy, the optimal timing of drug administration depends primarily on whether the compound inhibits primary nucleation, secondary nucleation or the growth of the aggregates. This strategy has also been tested in a model of Aβ overexpression in *C. elegans* [140].

Many other small molecules with the ability to act directly on the oligomers have also been tested in AD models in *C. elegans*. We will now give a brief description of them.

CNI-1493, a synthetic guanylylhydrazone developed as an inhibitor of arginine transport and nitric oxide production in macrophages, alleviated Aβ-induced behavioral deficits in a *C. elegans* model. Although this compound was initially used for its neuro-anti-inflammatory properties, the protective effect is probably mainly due to an anti-amyloidogenic mechanism mediated by direct interaction with Aβ [141].

The aminosterol trodusquemin increased aggregates formation while suppressing Aβ42-induced toxicity in a *C. elegans* model. The mechanism appears to be a displacement of the oligomers preventing their interaction with the membrane, and also an increased rate of conversion of the oligomers into less toxic fibrils [142].

Thioflavin, a dye traditionally used in histopathology to stain amyloid, extended the lifespan of a model of pathogenic mutated tau expression in *C. elegans*, much as it also did in the controls [143]. The effect could be mediated by an activation of the proteasome mediated by the induction of HSF-1 and SNK-1.

Analogues of the natural tubulin-binding compound combretastatin A-4 have also been studied. One of these analogues is PNR502. In transgenic strains of *C. elegans* expressing human Aβ42 in muscle or neurons, PNR502 rescued Aβ42-induced impairment of motility and chemotaxis [144].

Multitarget drugs have also been proposed with the idea of preventing the progression of AD by a synergistic mechanism. The compound used, 2-((1H-benzo[d]imidazol-2-yl)methoxy)benzoic acid (BIBA), consists of a group with an antiaggregating effect aimed at interacting with Aβ and an aspirin derivative with anti-inflammatory function. BIBA markedly prolonged longevity and alleviated Aβ-induced paralysis in transgenic *C. elegans* [145].

The 2-substituted 1,3-selenazole amide derivatives (CHF11) exhibited a protective effect against Aβ-induced toxicity in the *C. elegans* CL4176 model by reducing β-amyloid aggregation and ROS production [146].

Frondoside A is a saponin derived from the sea cucumber *Cucumaria frondosa* with anticancer activity, and also activity on Aβ aggregation and proteotoxicity. This compound, at low concentrations, significantly delayed paralysis in a *C. elegans* model with neuronal expression of Aβ [147]. Frondoside A mainly reduced the level of small oligomeric forms of Aβ, and also protected worms from oxidative stress and rescued chemotaxis dysfunction.

Another mechanism to block Aβ aggregation is photo-oxygenation. A supramolecular self-assembly targeting Aβ with switchable photoactivity has been designed and shown to be effective in preventing Aβ aggregation in vivo in the *C. elegans* CL2006 model [148].

By computational analysis of the human metabolome database, two endogenous metabolites, carnosine and kynuric acid, have been identified that inhibit Aβ aggregation and rescue alterations in the GMC101 model of AD in *C. elegans*. These metabolites act by triggering cytosolic UPR through the transcription factor HSF-1 [149]. *C. elegans* has also been used to investigate changes in the metabolome following induced expression of Aβ. The metabolomic study of an AD transgenic strain allowed the identification of changes in several metabolites previously associated with AD, such as high levels of allantoin and tyrosine, and decreased cystathionine, supporting the use of *C. elegans* as a model to study AD [150].

### 9.4. Modulators of Enzymes and Receptors Involved in AD

The enzymes responsible for O-linked N-acetylglucosamine (O-GlcNAc) cycling (OGT-1 and OGA-1 in *C. elegans*) play an important role in controlling cytoplasmic and nuclear proteostasis [151], and show high homology to human orthologues [152]. Mutants of these enzymes alter the toxicity of Aβ and mutant tau in *C. elegans* models [153], so it has been proposed to use these models to search for small-molecule modulators of O-GlcNAc metabolism that may influence neurodegeneration.

Alkaloids of *Lycoris radiata* delayed the paralysis of an Aβ-expressing transgenic strain of *C. elegans*, and the effect was mediated by the inhibition of the acetylcholinesterase gene expression [154].

Sig2R are chaperone proteins widely distributed in the central nervous system, and are involved in cellular processes related to cancer and neurodegenerative disorders. SAS-0132 and JVW-1009, Sig2R receptor antagonists, had a neuroprotective effect in a *C. elegans* model of APP overexpression [155].

A recombinant buckwheat trypsin inhibitor (rBTI) was able to extend lifespan and reduce paralysis triggered by Aβ toxicity in CL4176 AD worms, by a mechanism involving autophagy activation [156].

We have previously mentioned that knockout of the *dld-1* gene, which encodes for dihydrolipoamide dehydrogenase, has a protective effect in *C. elegans* models overexpressing Aβ [59,60]. An inhibitor of this enzyme, 2-methoxyindole-5-carboxylic acid (MICA), alleviated Aβ-induced paralysis and improved cholinergic neurotransmission in *C. elegans* expressing Aβ in muscle cells [59].

NP103, a selective inhibitor of GSK-3, the main kinase contributing to pathogenic tau hyperphosphorylation, ameliorated the paralysis phenotype of a mutant tau overexpression model in *C. elegans*, but not its lifespan [143].

Loss of function of the *C. elegans sul-2* gene (steroid sulfatase, STS) increased the concentration of sulfated steroid hormones, as well as longevity, and improved the phenotype in several *C. elegans* models of protein aggregation diseases. The same effects were obtained in *C. elegans* models using STX64, a specific STS inhibitor that also ameliorated the phenotype in a mammalian model of AD [157].

Cannabidiol increased longevity and prevented Aβ-induced neurite degeneration in a pan-neuronal Aβ expression model in *C. elegans* through a mechanism involving cannabinoid receptor 1 activation [158].

In relation to vitamin B12, it has been shown that if we feed *C. elegans* expressing Aβ with a diet deficient in vitamin B12, paralysis is accelerated, ATP levels are reduced and oxidative stress increases. The mechanism appears to be that vitamin B12, acting as a cofactor for methionine synthase, affects the methionine/S-adenosyl-methionine cycle and thus protects against Aβ-induced proteotoxicity [159]. Similarly, in the GMC101 strain expressing Aβ in muscle cells, worms lacking B12 supplementation showed more rapid and severe paralysis than those receiving it. B12 supplementation reduced homocysteine levels, suggesting that oxidative stress caused by elevated homocysteine levels may be an important factor in Aβ toxicity [160]. Consistently, betaine reduced Aβ-induced degeneration in an AD model in *C. elegans* through an activation of cystathionine-β-synthase and a reduction in homocysteine levels [161].

### 9.5. Other Compounds and Mechanisms Studied in C. elegans Models of AD

Resveratrol reduced paralysis in a *C. elegans* model of Aβ overexpression by a mechanism involving the activation of several proteins involved in proteostasis, including members of the unfolded protein response (UPR) pathways in mitochondria (UBL-5) and endoplasmic reticulum (XBP-1) [162]. Dauricin, an activator of XBP-1, also delayed the progression of paralysis in a model of Aβ expression in *C. elegans*, and the effect disappeared after *xbp-1* silencing [163]. Dauricin also increased the survival of the Aβ42 overexpression model GMC101 [164]. These data underscore the importance of UPR pathways in maintaining proteostasis.

Spermidine is an anti-aging small molecule whose molecular mechanism of action is the elimination of damaged mitochondria through the process of mitophagy. To evaluate the impact of spermidine, a *C. elegans* model with coexpression of human Aβ and tau proteins was used. Spermidine treatment resulted in a considerable increase in lifespan in this model [165].

Caffeic acid significantly alleviated Aβ-induced toxicity, increased longevity, decreased body paralysis, and ameliorated reproductive defects in the *C. elegans* model of Aß expression CL4176 [166].

Nicotine attenuated paralysis symptoms in several models of AD in *C. elegans*. Nicotine did not inhibit Aβ aggregation in vitro, but suppressed Aβ deposition and reduced Aβ oligomers to alleviate toxicity induced by Aβ overexpression in *C. elegans*. The effect was mediated by the SKN-1 pathway, which regulates detoxification processes and protection against stress [167].

One of the *C. elegans* models with Aβ expression in muscle showed that exercise produces an improvement in degeneration due to age and Aβ expression [168]. Prolonged swimming exercise training in *C. elegans* has also been described to improve health parameters such as mitochondrial respiration or survival, and to improve nervous system health by increasing learning capacity and protecting against neurodegeneration in models of tauopathy, Alzheimer’s disease and Huntington’s disease [169].

It has also been shown that replacing the *E. coli* OP50 commonly provided as food to the worm by Bacillus subtilis leads to the improvement of the paralysis phenotype and the restoration of longevity in *C. elegans* models expressing Aβ in muscle [170]. These data can be interpreted on the basis of the possible beneficial effects on AD of treatments that produce changes in the gut microbiota.

**Table 3:** Compounds used to alleviate toxicity in Alzheimer’s disease models in *C. elegans*.

Drug	Action	*C. elegans* AD Model	References
Metformin	Antidiabetic	Muscle Aβ (CL2006, CL4176) Pan-neuronal Aβ (CL2355, GRU102)	[45,46]
NT219	Antidiabetic	Muscle Aβ (CL2006)	[128]
Metformin + Lithium	Antidiabetic	Pan-neuronal Aβ (GRU102)	[129]
Clioquinol (8-hydroxyquinoline)	Antioxidant	Aβ in glutamatergic neurons (UA166)	[130]
MitoQ	Antioxidant	Muscle Aβ (CL2006)	[132]
diphenyldiselenide (PhSe)2	Antioxidant	Muscle Aβ (CL2006, CL4176) Pan-neuronal Aβ (CL2355)	[133]
Antibodies anti-Aβ	Anti-amyloidogenic	Muscle Aβ (GMC101)	[134]
Humanin peptide	Anti-amyloidogenic	Muscle Aβ (CL4176)	[135]
Lactoferrin-derived peptides	Anti-amyloidogenic	Muscle Aβ (CL4176)	[136]
Bicyclic peptides	Anti-amyloidogenic	Muscle Aβ (GMC101)	[137]
Peptides from *C. ternatea*	Anti-amyloidogenic	Muscle Aβ (CL4176). Pan-neuronal Aβ (CL2355)	[138]
CNI-1493 and C1213	Anti-amyloidogenic	Muscle Aβ (CL2006, CL4176)	[141]
Aminosterol trodusquemin	Anti-amyloidogenic	Muscle Aβ (GMC101)	[142]
Thioflavin	Anti-amyloidogenic	Tau-V337M (aex-3/T337)	[143]
PNR502 (tubulin binding compound)	Anti-amyloidogenic	Muscle Aβ (CL4176). Pan-neuronal Aβ (CL2355)	[144]
BIBA (antiaggregating + anti-inflammatory)	Anti-amyloidogenic	Muscle Aβ (CL4176)	[145]
CHF11	Anti-amyloidogenic	Muscle Aβ (CL4176)	[146]
Frondoside A	Anti-amyloidogenic	Muscle Aβ (CL2006, CL4176). Pan-neuronal Aβ (CL2355, GRU102)	[147]
Photo-oxygenation	Anti-amyloidogenic	Muscle Aβ (CL2006)	[148]
Carnosine and kynuric acid	Anti-amyloidogenic	Muscle Aβ (GMC101)	[149]
Alkaloids of *Lycoris radiata*	Acetylcholinesterase gene expression inhibition	Muscle Aβ (CL4176)	[154]
SAS-0132 and JVW-1009	Sig2R antagonists	Pan-neuronal APP overexpression	[155]
Buckwheat trypsin inhibitor	Autophagy activation	Muscle Aβ (CL4176)	[156]
MICA	Dihydrolipoamide dehydrogenase inhibitor	Muscle Aβ (CL2006, CL4176) Pan-neuronal Aβ (CL2355)	[59]
NP103	GSK-3 inhibitor	Tau-V337M (*aex-3*/T337)	[143]
STX64	Steroid sulfatase inhibitor	Muscle Aβ (CL2006, GMC101)	[157]
Cannabidiol	Cannabinoid receptor 1 activation	Pan-neuronal Aβ (CL2355)	[158]
Vitamine B12	Methionine synthase activation	Muscle Aβ (CL4176, GMC101)	[159,160]
Betaine	Cystathionine-β-synthase activation	Muscle Aβ (CL2006)	[161]
Resveratrol	Mitochondrial and ER UPR activation	Muscle Aβ (CL2006)	[162]
Dauricin	ER UPR activation	Muscle Aβ (CL2120, GMC101)	[163,164]
Spermidine	Mitophagy activation	Pan-neuronal Aβ and tau overexpression (UM0001)	[165]
Caffeic acid	Antioxidant, antiaggregating	Muscle Aβ (CL4176)	[166]
Nicotine	SKN-1 pathway activation	Muscle Aβ (CL4176, CL2120)	[167]
Swimming exercise	Antioxidant, others	Muscle Aβ (CL2120). Pan-neuronal Aβ (CL2355)	[168,169]
Bacillus Subtilis	Gut microbiota	Muscle Aβ (CL2120, GMC101)	[170]

Abbreviations: ER, endoplasmic reticulum; UPR, unfolded protein response.

## 10. Conclusions

*C. elegans* has become an essential model for studying the molecular mechanisms of AD and for the search for new treatments. Models of Aβ and tau expression have provided very relevant information on their mechanisms of toxicity and on the possible enhancers and suppressors of this toxicity. The wide range of genetic tools and functional studies available allow a very detailed characterization of the effects of each gene variant or neuroprotective compound. It should be noted that in many cases *C. elegans* data have already predicted similar effects in other models of neurodegeneration or in human AD. *C. elegans* models are also excellent for the high-throughput screening of potential drugs, and this strategy promises to be very effective for the identification of new therapeutic targets.

In any case, we must also be aware of the limitations of the model. *C. elegans* lacks many mammalian organs, including the circulatory system. The nervous system is very simple, lacks myelinated neurons and can in no way simulate the complexity of the human nervous system. The absorption and distribution of drugs is hampered by its hard cuticle and the absence of a circulatory system. Like all models, therefore, it has advantages and limitations, so the data obtained must pass through the filter of validation in other models before being applied to human AD. However, the ability of *C. elegans* models to perform rapid and simple studies with a large number of individuals and at low cost represents a huge advantage for initial studies or the massive search for new compounds.

## Figures and Tables

**Table 1:** Correspondence between human genes associated with AD and their *C. elegans* orthologues.

Human Genes Associated with AD [13]	*C. elegans* Orthologue [12]	FAD/LOAD
Amyloid Beta Precursor Protein (*APP*)	*apl-1*	FAD
Amyloid Beta Precursor-Like Protein 1 (*APLP1*)	*apl-1*	-
Amyloid Beta Precursor-Like Protein 2 (*APLP2*)	*apl-1*	-
α-secretase (*ADAM10* and *ADAM17*)	*sup-17 and adm-4*	LOAD
β-secretase (*BACE1*)	*none*	-
γ-secretase complex, Presenilin 1 (*PSEN1*)	*sel-12, hop-1 and spe-4*	FAD
γ-secretase complex, Presenilin 2 (*PSEN2*)	*sel-12, hop-1 and spe-4*	FAD
γ-secretase complex, Nicastrin (*NCSTN*)	*aph-2*	-
γ-secretase complex, Anterior pharynx-defective-1 (*APH1A*)	*aph-1*	-
γ-secretase complex, Presenilin enhancer 2-subunit (*PSENEN*)	*pen-2*	-
Microtubule associated protein *MAP2/MAP4/MAPT*/Tau	*ptl-1*	LOAD
Apolipoprotein E, *APOE*	*none*	LOAD
Glycogen synthase kinase 3 beta, *GSK3β*	*gsk-3*	LOAD
Phosphoinositide-binding clathrin adaptor, domain 2 (*SNAP91, PICALM*)	*unc-11*	LOAD
Bridging integrator 1, Amphiphysin family (*BIN1, BIN2, AMPH*)	*amph-1*	LOAD
Clusterin-associated protein-1 (*CLUAP1*)	*dyf-3*	LOAD
Ephrin Type-A Receptor 1 (*EPHA1*)	*vab-1*	LOAD
Clusterin (*CLU/TRPM2*)	*ced-11*	LOAD
Inositol Polyphosphate-5-Phosphatase D (*INPP5D*)	*inpp-1*	LOAD
Complement component receptor 1 (*CR1*)	*lev-9*	LOAD
ABI Family Member 3 (*ABI3*)	*abi-1*	LOAD
Phospholipase Cγ2 (*PLCG2*)	*plc-3*	LOAD
Myocyte-Specific Enhancer Factor 2C (*MEF2C*)	*mef-2*	LOAD
CD2-Associated Protein (*CD2AP*)	*Y44E3A.4*	LOAD
Nuclear Polyadenylated RNA-Binding Protein (*CELF1*)	*etr-1*	LOAD
PH Domain-Containing Family C1 (*FERMT2*)	*unc-112*	LOAD
Thioredoxin Domain-Containing Protein (*NME8*)	*ndk-1*	LOAD
Sortilin Related Receptor 1 (*SORL1*)	*F14B4.1*	LOAD
Phospholipid-Transporting ATPase (*ABCA7*)	*abt-2*	LOAD
Sodium/Potassium/Calcium Exchanger (*SLC24A2/A4*)	*ncx-4, ncx-5*	LOAD
Ras Additionally, Rab Interactor 3 (*RIN3*)	*rin-1*	LOAD
Protein Tyrosine Kinase 2 Beta (*PTK2B*)	*kin-32*	LOAD
Enoyl-CoA Hydratase Domain Containing 3 (*ECHDC3*)	*ech-2*	LOAD
Angiotensin I Converting Enzyme (*ACE*)	*acn-1*	LOAD
A Disintegrin Additionally, Metalloproteinase With Thrombospondin Motifs 1 (*ADAMTS1*)	*gon-1*	LOAD
Thyroid Hormone Receptor Interactor 4 (*TRIP4*)	*asc-1*	LOAD
Retinoic Acid Receptor-Related Orphan Receptor A (*RORA*)	*nhr-23*	LOAD
Zinc Finger Protein 423 (*ZNF423*)	*lin-13*	LOAD
Zinc Finger Protein 655 (*ZNF655*)	*ztf-2*	LOAD
Benzodiazepine Receptor-Associated Protein 1 (*TSPOAP1, BZRAP1*)	*rimb-1*	LOAD
Trophoblast Glycoprotein (*TPBG*)	*lron-3*	LOAD
Heparan Sulfate-Glucosamine 3-Sulfotransferase 1 (*HS3ST1*)	*hst-3.1*	LOAD
Protein Kinase D3 (*PRKD3*)	*dkf-2*	LOAD
NADH:Ubiquinone Oxidoreductase Complex Assembly Factor 7 (*NDUFAF7*)	*ZK1128.1*	LOAD
Nicotinic acetylcholine receptor Epsilon Subunit (*CHRNE*)	*acr-2, acr-3, acr-6, acr-8, acr-12, lev-1, lev-8, unc-29, unc-38, unc-63*	LOAD
Repressor element 1-silencing transcription factor (*REST*)	*spr-3* and *spr-4*	LOAD

Abbreviations: AD, Alzheimer’s disease; FAD, familial AD; LOAD, late-onset AD.

**Table 2:** Pros and cons for usage of *C. elegans* models to study pathological processes in AD.

Pros
Many human genes possess orthologues in *C. elegans*, among them most (but not all) of the genes involved in Alzheimer’s disease
Aβ expression affects similar pathways in worm, mouse and human
Short generation and life cycle, around 3 weeks, and low maintenance and propagation costs
Small nervous system, only 302 neurons, with an invariant neuronal network
Transparent body, allows visualization of fluorescent proteins at all stages of its life
Complete characterization of cell fate lineage and neuronal connectivity
Complete genome sequence and very powerful genetic manipulation tools
Wide availability of mutant strains of most of the genes
Availability of extensive RNAi libraries able to silence most of the genes
Conserved protein interaction networks involved in AD
Numerous methods available for the functional characterization of neurodegeneration, motility disturbances or protein aggregation
Ability to make high throughput chemical screens for drug assay
**Cons**
Lacks β-secretase and β-amyloid peptide sequence in APP. Unable to generate endogenous Aβ
Lacks *APOE* gene
Lack of many specific mammalian features: circulatory system, myelinated neurons, defined brain structures such as hippocampus or cortex, complex connections of the human brain, adaptative immune system, among others
